# Design Time Optimization for Hardware Watermarking Protection of HDL Designs

**DOI:** 10.1155/2015/752969

**Published:** 2015-03-15

**Authors:** E. Castillo, D. P. Morales, A. García, L. Parrilla, E. Todorovich, U. Meyer-Baese

**Affiliations:** ^1^Department of Electronics and Computer Technology, University of Granada, Campus Universitario Fuentenueva, 18071 Granada, Spain; ^2^Instituto de Investigación en Tecnología Informática Avanzada (INTIA), Universidad Nacional del Centro de la Provincia de Buenos Aires (UNCPBA), B7001BBO Tandil, Argentina; ^3^Department of Electrical and Computer Engineering, Florida State University (FSU), 2525 Pottsdamer Street, Tallahassee, FL 32310, USA

## Abstract

HDL-level design offers important advantages for the application of watermarking to IP cores, but its complexity also requires tools automating these watermarking algorithms. A new tool for signature distribution through combinational logic is proposed in this work. IPP@HDL, a previously proposed high-level watermarking technique, has been employed for evaluating the tool. IPP@HDL relies on spreading the bits of a digital signature at the HDL design level using combinational logic included within the original system. The development of this new tool for the signature distribution has not only extended and eased the applicability of this IPP technique, but it has also improved the signature hosting process itself. Three algorithms were studied in order to develop this automated tool. The selection of a cost function determines the best hosting solutions in terms of area and performance penalties on the IP core to protect. An 1D-DWT core and MD5 and SHA1 digital signatures were used in order to illustrate the benefits of the new tool and its optimization related to the extraction logic resources. Among the proposed algorithms, the alternative based on simulated annealing reduces the additional resources while maintaining an acceptable computation time and also saving designer effort and time.

## 1. Introduction

The methodologies based on reuse of IP components [1–3] critically depend on improved techniques to protect IP ownership. This is due to the rapidly growing international trade in stolen intellectual property (IP), counterfeit chips, and cloned designs. The current technology offers new intellectual property protection (IPP) mechanisms, with fingerprinting and watermarking being the options that incite the greatest interest [4–6]. The principal focus of watermarking techniques for IP cores is to enable the protection of author rights in the development and distribution of IC designs as reusable modules. Several watermarking techniques for IP cores have been proposed [7–9]. These IPP watermarking techniques can be classified in several ways. One of these classifications distinguishes between additive-based [4, 10] and constraint-based techniques [11, 12]. The fundamental idea of additive watermarking is to add something to a design, which would not be present normally, yet is hard to detect and would ideally damage the design if removed. Bai et al. [10] propose to obtain the signature at the output ports by triggering the added circuit through feeding a special signal sequence to the inputs. General constraint-based watermarking techniques encode an author's signature into an optimization or synthesis problem, by limiting the overall solutions space. This approach major drawback is their limitation on watermarked core verification options. New research lines are based on power side channel in order to ease the signature extraction for embedded IP cores [13–15]. On the other hand, other possible classifications of IPP watermarking techniques are based on the type of IP core that requires protection or the design level of the watermark application [16, 17]. In particular, IPP watermarking techniques have been applied to hardware description language (HDL) IPs [17–20]. The embedding of a watermark at this design level is highly tamper-resistant, since the signature is embedded in preliminary stages and is therefore dragged through the whole design flow. To prevent reverse engineering on the distribution of the protected high-level design, encryption and obfuscation techniques can be applied [21, 22]. Obfuscation techniques consist in hiding the design concept or program algorithm included in the source by using one or more transformations of the original code.

This work presents a new tool for signature insertion at behavioral level. The tool distributes the bits of a digital signature through the combinational logic included in the design to protect and can be combined with different methods for signature extraction activation and for signature verification. This tool has been applied for the signature distribution process of IPP@HDL [17] in order to automate and ease this process, although its applicability can be extended to other HDL watermarking methods. This work also includes an analysis and evaluation of the different algorithms developed for signature distribution and insertion.

The remainder of this paper is organized as follows: Section 2 describes the processes involved in the HDL watermarking techniques and analyzes the methods proposed in the literature, summarizes the most relevant aspects of IPP@HDL and the underlying ideas for the signature distribution tool, focuses on several important considerations for signature distribution, describes the developed insertion algorithms, and also includes a detailed evaluation of these signature insertion algorithms.  Section 3 analyzes implementation results including the use of the automated algorithms for the IPP@HDL application, and, finally, Section 4 summarizes the main conclusions.

## 2. Materials and Methods

### 2.1. High-Level Watermarking Background

**Figure 1:**
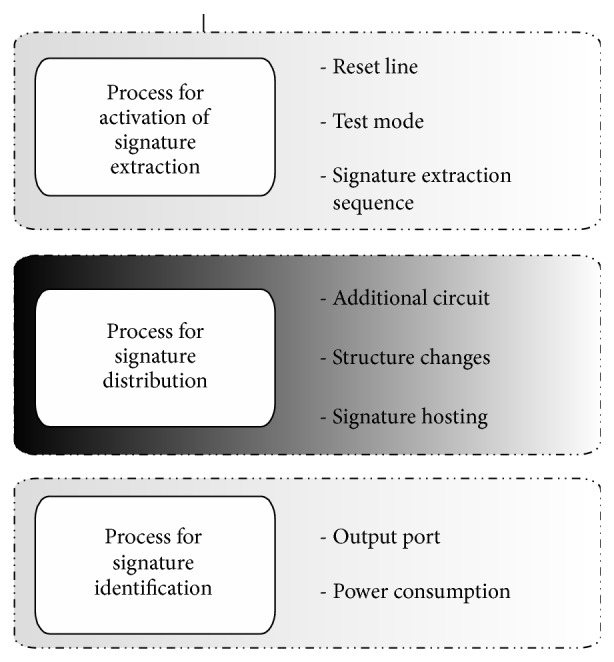
Processes involved in HDL watermarking.

HDL watermarking techniques allow protection to be carried out over the design and implementation processes when the designer has absolute control over the design, which is not possible to be regained for a finalized IP core. Thus, this design level offers important advantages over physical and synthesis levels for managing the application of the watermarking techniques, since the watermark could be embedded as a functional part of the design. However, there are some aspects of the related works that should be analyzed. The development of any watermarking technique is based on the application of three processes, as Figure 1 shows:

- process for activation of the signature extraction;
- process for signature distribution;
- process for signature identification.

Different options for the activation of signature extraction exist. Some of the related works propose activation to be always enabled, activation using the reset line [23] or the test mode [24], or activation by means of a signature extraction sequence [10, 17] (in some cases using input pins of the IPP core). Other trends make use of output pins of the core under protection to extract the digital signature [17]. More recent research lines utilize measurements of power consumption of the system to recover the contents of the embedded signature by means of the DC consumption analysis of the overall system [13–16, 23]. Through an exhaustive analysis of the current HDL watermarking methods for signature distribution, at least three different strategies can be distinguished. One of these is based on the design of an additional circuit, which contains the digital signature and is integrated into the original core [20, 24]. This strategy allows easy application but provides low security levels, since the independent module containing the information identifying the IP core could be removed without affecting the correct functioning of the original IP core. This drawback was addressed by Yu and Zhu [25], but neither automation nor computation times are shown for the proposed method. Other strategy is based on changes into the original structure of the design [19, 26]. Yuan et al. [26] propose several schemes for HDL code protection. However, some of the experimental results show that the information they embed as watermark is only 56 bits as maximum, offering low proof of authorship when compared to other watermarking strategies [17, 19]. In addition, for some benchmark circuits, the area overhead is high. Other techniques of this type are based on FSM watermarking [19, 27]. Concretely, Charbon and Torunoglu [19] specify sequential circuits as finite state machines (FSMs), minimize the FSMs, and embed the digital signature as nonspecified IO mapping. The experimental results show low CPU times for the proposed Monte Carlo approach but the area overhead is unacceptable for some of the FSM benchmarks used as example. In addition, the approach is limited to having a sequential circuit specified as FSM or to transforming it to a FSM, conserving the functioning of the original design, but changing completely the HDL specification and the structure for the implementation of the original design.

The third category for HDL watermarking techniques is based on the processes for signature distribution. The difference between hosting strategies and those based on structure changes lies in the fact that when hosting is applied, no changes are made to the original design. The signature hosting strategy proposed in IPP@HDL [17] showed that it is possible to look for or to identify blocks of the signature bits within the output patterns of the combinational logic included in the design, independently of the logic structure used for its implementation (look-up, logic gate networks, etc.). This strategy also offers high resistance against attacks as it is detailed in the exhaustive study included in [17]. Drawbacks are related to design effort and time for its application.

The different methods for signature distribution, signature extraction activation, and signature identification can be combined in order to develop a robust and solid HDL watermarking technique. However, the objective of the proposed work is only the study and improvement of the methods for signature distribution.

The most relevant objective criteria necessary for evaluating an HDL watermarking technique are correctness of functionality, hardware overhead, proof of authorship, and resistance against attacks [17]. Other important metrics, usually not considered for real evaluation, are related to the transparency and the complexity of the processes and the design time and effort required for their application [17]. Some of these metrics, especially design time and effort, can be crucial for the practical applicability of a given protection method. Thus, in the application of any watermarking technique it is very important to have in mind these criteria and to try to develop a strategy getting a positive evaluation. To get this objective, every one of the processes implied in the watermarking strategy is very important, but, as it was commented above, the proposed work is focused on the signature distribution and insertion process for HDL watermarking techniques. From the analysis of the methods for signature distribution, it is possible to extract important information about the evaluation metrics. Security, area overhead, and design time and effort are usually inversely related. Thus, a solid method for signature distribution should require reduced design time and effort and offer high security while the impact of its application over the original design should imply low area overhead. This work tries to reach these objectives using one of the watermarking techniques analyzed above, IPP@HDL hosting strategy [17]. This watermarking technique offers important advantages but needs a designer's time reduction, while the original advantages, namely, reduced area overhead and high security level, are maintained or improved. The main problem for the designer is to find an easy, fast, and optimized form to distribute the bits of the signature. Thus, this paper proposes efficient new mechanisms to ease signature distribution and insertion and to reduce the associated time and effort. These advances in automation and optimization of IPP@HDL contribute to considerably reduce design time and effort for the application of this watermarking technique to a specific IP core. In addition, the new tool for signature distribution also makes it possible to easily select the best signature spreading option for reducing the overhead area of the protected IP core. The proposed advances are not only useful for IPP@HDL, but this new tool can be extended to any HDL watermarking technique based on the distribution of signature bit blocks through combinational logic included in the design.

### 2.2. IPP@HDL Overview

#### 2.2.1. IPP@HDL Signature Hosting Strategy

**Figure 2:**
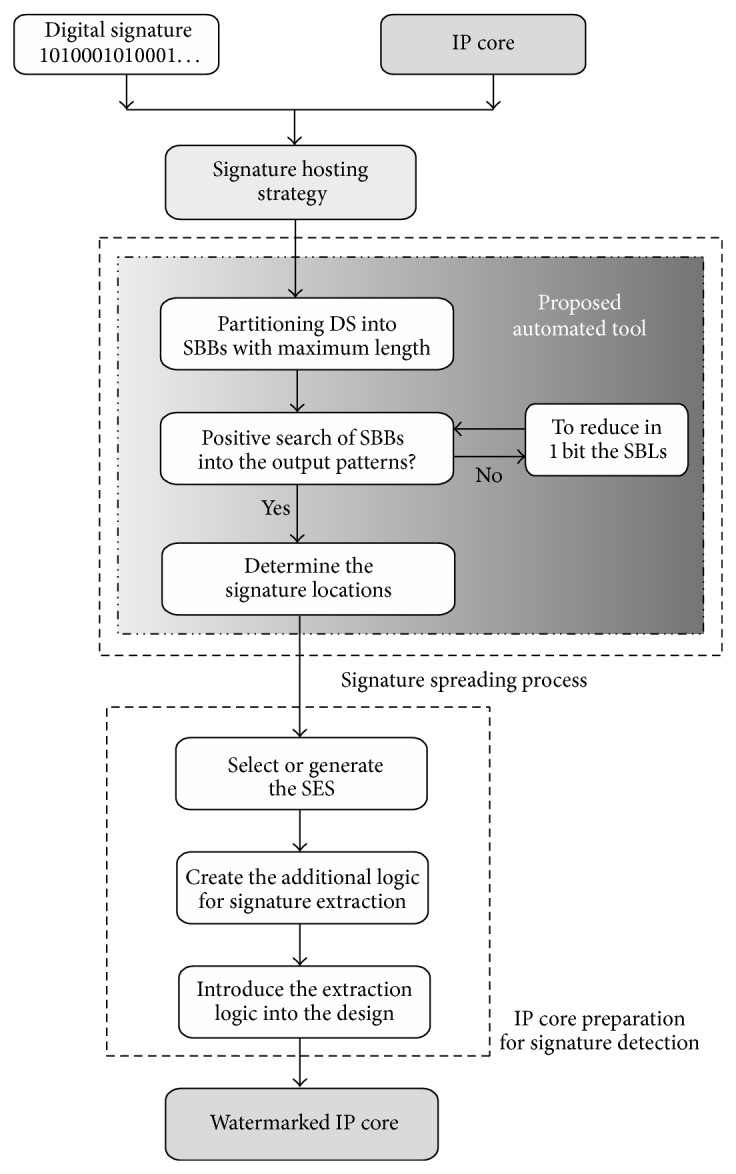
Generation of the watermarked IP core using IPP@HDL.

IPP@HDL signature hosting strategy [17] protects digital systems by spreading the bits of a digital signature [28] through combinational logic included in the high-level description of the design [17, 29]. The digital signature is propagated through the whole design flow down to the physical implementation, alleviating the requirement of additional system resources. Thus, the system is protected from the first stages of logic synthesis without requiring any resynthesis, while it keeps this protection through place and route for whatever target technology is considered (ASIC, FPGA, etc.); therefore, the final physical implementation is also protected for its distribution. In addition, IPP@HDL includes an easy and secure automated procedure for nondestructive signature detection, requiring minimal hardware to be included in the system [17]. These resources will process the petition for signature extraction by detecting a specific input sequence, called signature extraction sequence (SES), and will generate the signature positions and route the signature bits as a data sequence to the output of the protected system.  Figure 2 shows the processes for the application of IPP@HDL. The system receives the original IP core and the digital signature and generates the watermarked IP core. This figure also shows how the automated tool is integrated into this IPP strategy and its main tasks, which will be analyzed in Section 2.3.

#### 2.2.2. Evaluation of IPP@HDL

The following criteria were defined to evaluate IPP@HDL: implementation and hardware issues, signature strength, resistance against attacks, SES invulnerability, and proof of authorship. Synthesis data [17] show that IPP@HDL results in negligible degradation of system performance and very low area penalties for a variety of digital signatures and signature extraction hardware options. Also, the attack analysis and the probabilistic studies showed that IPP@HDL is a secure method and provides high SES invulnerability, as well as strong proof of authorship. Thus, the experimental results and the exhaustive analysis of the established criteria made it possible to evaluate positively IPP@HDL as IPP watermarking technique. The novelty introduced by IPP@HDL signature hosting strategy is that it makes the signature bits part of the original design, while the original design is not modified by adding or embedding signature bits. This strategy increases the attack resistance, since trying to remove or change a single bit would result in an incorrect circuit functioning, while the system itself extracts the signature bits when it is required to do so. In addition, the comparison with previous IPP techniques and the summary of the new contributions of IPP@HDL reveal the strength and the important advantages of this proposal. Among these advantages, IPP@HDL allows introducing digital signatures obtained with cryptographic tools. Thus, it allows the prevention of different types of assaults and these signatures being larger than the usually employed ones in the majority of the design examples available for other watermarking techniques [24, 26].

#### 2.2.3. Advances and Improvements

The signature extraction procedure proposed in IPP@HDL presumes that the I/O pins of the core under protection are accessible in order to activate the signature extraction process and for the recovering of the signature. However, in embedded cores, the only* a priori* accessible pin is the reset line. To overcome this, other extraction procedures could be used in IPP@HDL to activate the signature extraction for achieving the protection of an isolated core embedded in a complex system. Some works have studied new alternatives for the signature extraction process based on power analysis [15, 23]. A method to easily integrate the watermark into a core and easily extract this watermark over the power pins of the chip is proposed in [15]. On the other hand, a new method for extracting digital signatures from watermarked protected cores that are embedded into complex systems, without direct access to the I/O pins, has been recently proposed in [23]. The technique makes use of the reset line for signature extraction activation and a procedure based on the variation of power consumption for the signature identification. This signature extraction method is applied using the IPP@HDL general procedure and extends the procedure aiming at the extraction of the signature without direct access to the I/O pins of the core under protection. This extension, named e-IPP@HDL, activates the extraction of the digital signature using only the reset line and recovers by itself the contents of this signature through the DC consumption analysis of the overall system. The presented results show a low area impact with high performance and reliability. This new activation of the signature extraction forms part of different alternatives to be used in IPP@HDL, offering the mentioned advantages. Since the objective of this work is not the activation of the signature extraction, but just the automation of the distribution and insertion of signature bits independently of other aspects of the watermarking process, the procedure presented in the following sections can be combined with any other variant of IPP@HDL or even applied to other similar watermarking strategies, as stated above. To secure the HDL code describing the watermarked design that will be delivered to a customer, encryption or obfuscation tools [22] may be used after IPP@HDL application. Recently, a set of open source obfuscation tools has been developed, which allows very long, hard to read identifiers to be used [21]. Comment methods that allow adding copyright and limited warranty information are also implemented. Open source C, VHDL, and Verilog tools have been developed and tested for Altera and Xilinx tools and devices. These proposed methods for code obfuscation seem a viable IPP method given the small penalty and good success on avoiding reverse engineering.

### 2.3. New Tool for Signature Hosting

A more exhaustive analysis and evaluation than the one presented in [17] may be carried out for signature distribution proposals. For the IPP@HDL signature hosting strategy the signature blocks are hosted by looking for output patterns of combinational logic that are equal to the signature blocks. The search method for these signature blocks depends on each given design and digital signature, requiring substantial time and effort. In addition, different options for hosting these blocks can be considered. The best hosting option is the one requiring less effort and introducing less area and performance overhead. Usually, to manually consider all the hosting solutions and to locate the best one are difficult, if not impossible, and it also requires considerable designer effort and time. From this analysis, some new advances and improvements for the signature hosting strategy are proposed in this work. Thus, a software tool has been developed for automated hosting purposes. Its main objectives are

1. to automate the signature hosting process in order to save time and effort;
2. to select the signature block length for optimization purposes;
3. to select a cost function that determines the best hosting solution for the IP core to be protected in terms of area and performance penalties;
4. to select a hosting algorithm that reduces this cost function and requires assumable computation time.

#### 2.3.1. Signature Hosting Automation

The first objective of the new tool is to reduce time and effort for the signature hosting process. As in a manual search, the automated tool performs a search for the signature bit blocks, identifying them within output patterns from the combinational logic included in the original IP core. The software tool can perform the search process in two ways: considering the truth tables of the combinational logic or reproducing the operations that the combinational logic implements. As a result of this search, the automated tool then generates an input pattern set, signature locations (SLs), which addresses the output patterns where the signature bit blocks are identified.

#### 2.3.2. Signature Block Length

For IPP@HDL, the best signature hosting option is the one requiring a minimum of additional resources for signature extraction logic. Obviously, signature blocks to be hosted are required to have a bit length equal or minor to that of the output patterns that will host them. The selection of the most appropriate signature block length (SBL) will depend on each given design and its signature bits. Assuming a *n*-bit digital signature partitioned into *m* blocks of *b* bits, *NC*-bit output patterns for the combinational logic included in the original IP core, and *C* ≥ *b*, the next equation shows the hosting probability, that is, the probability of identifying each one of the *m* signature blocks of *b* bits with at least one of the *N* output patterns:

(1) $$\begin{array}{c} P_{\text{hosting}}={\left(1-{\left(\frac{2^{b}-1}{2^{b}}\right)}^{N}\right)}^{m}. \end{array}$$

It is possible to reduce the implementation resources of the signature extraction hardware, an FSM [17]. This is the case if the SBL is equal to the output pattern size; that is, *b* = *C*, since the number of signature blocks, *m*, is the lowest. Thus, the number of FSM states is as small as possible, contributing to reduce the required additional resources. However, if an evaluation of (1) for different SBLs is made, the hosting probability for *b* = *C* is the lowest. On the other hand, small lengths for signature blocks increase the hosting probability but, in these cases, the additional resources for extraction logic also increase: as the SBL size decreases, the number of signature bit blocks to be hosted increases, and this implies more states for the extraction logic and, consequently, more area overhead for the watermarked IP core. Thus, it is established as first criteria for the automated tool that it will begin the signature block search considering lengths equal to that of the output patterns of the combinational logic selected for identifying the signature blocks; that is, *b* = *C*. In case this search results in a failed attempt for any of the signature blocks, the automated tool initiates the search reducing by 1 bit the SBL size and so on, until all blocks have been identified. One of the most important advantages of the automatic tool over the manual hosting is that it allows considering different lengths for the signature blocks and finds a solution that allows full signature hosting while minimizing extraction logic resources. With adequate automated processes, this should not require excessive time and effort, as it will be shown later.

#### 2.3.3. Cost Function

Once the automated tool has selected the larger SBL allowing full signature hosting, there are some others aspects that need to be analyzed. An important fact to be considered is that, for a given SBL and signature block, it is possible to find different input patterns that generate the same output and where this signature block could be hosted. Thus, for every signature block, the automatic tool should also select one option among all the possible input patterns and establish it as the corresponding signature location (SL). The simplest option is to make the selection in a random way; so this was the approximation considered for developing the first automated hosting algorithm. However, it is possible to take advantage of the different possibilities for hosting each signature block and to select the option optimizing the implementation resources required by the signature extraction logic. Once again, if the architecture of the signature extraction hardware [17] is considered, the Hamming distance between the input patterns that will generate the signature blocks can be selected as cost function. The objective is to reduce as much as possible the signal transitions used by the FSM included in the signature extraction logic. It is thus possible to reduce the combinational logic required for the FSM [30] if the hosting solution minimizes the transitions between these patterns that the FSM feed to the signature locations. As it was commented above, this extraction logic takes the input patterns selected by the automated tool as signature locations, uses them to address the signature blocks, and routes these blocks to the signature extraction system.

### 2.4. Algorithms for Signature Distribution

Three different algorithms were developed for signature hosting [31], which are detailed below.

#### 2.4.1. Random Location Assignment (RLA) Algorithm

The first algorithm, called Random Location Assignment algorithm, is based on a random decomposition of each one of the signature blocks. No cost function is considered for this algorithm, and no optimization for additional extraction logic is carried out. It is very similar to a manual search without optimization, but the process is automated and thus allows saving considerable effort and time. On the other hand, besides being the simplest algorithm, its major advantage is the computation time, since for each signature block the search stops when the first hosting solution is found.

#### 2.4.2. Exhaustive Paired Optimized (EPO) Algorithm

This algorithm is based on a search of the minimum Hamming distance by means of an exhaustive search between the decomposition of every signature block and the adjacent one. The first two blocks are adjusted simultaneously, selecting as a solution the one that minimizes the Hamming distance between the corresponding input patterns. The remaining signature blocks are adjusted performing a search for the input pattern/s with minimum Hamming distance to the input pattern/s for the previous signature block, fixed in the previous step. Thus, to obtain the optimum solution for inserting a signature block, all the hosting solutions are searched and the algorithm selects the one minimizing the Hamming distance with the hosting solution of the previous signature block. This algorithm does not achieve the optimum possible solution, since not all the signature blocks are considered simultaneously. However, the total Hamming distance between all the signature block decompositions is close to the minimum global Hamming distance. The main drawback of this algorithm is the computation time, since it increases exponentially with the SBL. As it will be shown in Section 2.5, for certain block lengths, the computation time becomes unaffordable.

#### 2.4.3. Simulated Annealing (SAMB) Algorithm

The third proposed algorithm for signature hosting concentrates on using a semiheuristic search through a simulated annealing (SA) algorithm [31]. SA was introduced more than twenty years ago, and since then it has been considered a useful tool for a variety of optimization problems in various fields [32–34]. The main feature provided by SA over other algorithms is its ability to avoid local minima by controlling the acceptance of cost-increasing neighbours by means of a probability, which decreases as the search algorithm progresses. Thus, a particular solution involving an increase in the cost function is accepted with probability:

(2) $$\begin{array}{c} P\left(\delta \right)=e^{\left(-\delta /t\right)}, \end{array}$$

where *δ* is the cost difference and *t* is the control parameter corresponding to the temperature in the physical analogy [35], so it is usually called temperature. The algorithm requires a careful adjustment in order to avoid high execution times [32] while maintaining the quality of the solution.

In the context of application to IPP@HDL automation, the proposed algorithm, called SAMB, globally minimizes the Hamming distance between adjacent signature blocks and optimizes the solution for each signature block using a SA process, taking into account the hosting solution for all the signature blocks. As it will be shown below, compared to the EPO algorithm, this SAMB algorithm reduces the computation times for large SBL sizes. Thus, SAMB gets to overcome one of the barriers of using heuristic algorithms related to high computing time, which is not easy to get over. In this algorithm the solution space includes all possible combinations of input patterns that lead to spreading the signature bit blocks. The initial solution is properly chosen to explore as best as possible the solution space. On the other hand, the selected cost function is the same as for the EPO algorithm, the Hamming distance between input patterns that generate two adjacent blocks of the digital signature. Other parameters or decisions were defined, taking into account the combinational logic used for signature hosting or experimentally adjusted trying to limit computation times.  Algorithm 1 shows the general scheme proposed for this SA algorithm.

As examples to initially illustrate the SA algorithm for IPP@HDL, two independent combinational logics for the decomposition of each block of a digital signature were considered. These combinational circuits are the sum and the linear combination. Let *m* be the number of signature blocks, and *B*_*i*_(*i* = 1,2,…, *m*) each one of these signature blocks. The decomposition of each signature block, for the sum and the linear combination, can be expressed as

(3) $$\begin{array}{c} B_{i}=S_{i1}+S_{i2}, \end{array}$$

(4) $$\begin{array}{c} B_{i}=P_{i1}+P_{i2}=a_{i}\times b_{i}+c_{i}\times d_{i}. \end{array}$$

For both combinational logics, the initial state for the decomposition of the first signature block, *B*_1_, is the half value of the data to decompose. In addition, for the linear combination, *P*_11_ and *P*_12_ have to be decomposed, as (4) shows. The iterative process to achieve this decomposition is described as follows:

1. initially set *a*_*i*_ = 1, *c*_*i*_ = 1;
2. test if for *a*_*i*_ = 1 and *c*_*i*_ = 1 it is possible to decompose *P*_11_ and *P*_12_; it is necessary to verify if the values of *b*_*i*_ and *d*_*i*_ required for the decomposition are enabled or included in the possible range;
3. if verification of step (2) is positive, the decomposition process for block *B*_1_ is finished;
4. if verification of step (2) is negative, *a*_*i*_ and *b*_*i*_ are increased by 1, and the algorithm returns to step (2).

For both combinational circuits, there is no next state for the decomposition of the first signature block, *B*_1_. For the remaining signature blocks (*B*_*i*_, *i* = 2,…, *m*), the initial state is *S*_*i*1_ = *S*_*i*−1,1_, *S*_*i*2_ = *B*_*i*_ − *S*_*i*1_, for the sum, and *P*_*i*1_ = *P*_*i*−1,1_, *P*_*i*2_ = *B*_*i*_ − *P*_*i*1_ for the linear combination. In case *B*_*i*_ < *S*_*i*−1,1_, the initial state for the block *B*_*i*_ considering the sum is *S*_*i*1_ = 0 and *S*_*i*2_ = *B*_*i*_, and the initial state for the block *B*_*i*_ considering the linear combination is *P*_*i*1_ = 0*P*_*i*2_ = *B*_*i*_. The value *S*_*i*1_ or *P*_*i*1_, or the next state, is calculated by randomly adding or subtracting 20%, and then the values *S*_*i*2_ or *P*_*i*2_ for this new state are computed. For the linear combination, *P*_*i*1_ and *P*_*i*2_ have to be decomposed using the iterative process described above. The validation of the new state is carried out as Algorithm 1 shows. The process continues reducing the temperature progressively until it reaches the stop criterion.

### 2.5. Algorithm Analysis

The proposed automated algorithms for signature hosting were analyzed and compared. For this comparison, different combinational logics were considered. As in the example detailed in the section above, the sum and the linear combination have been selected as examples of combinational logics for independent signature hosting. In addition, random series of MD5 and SHA1 [17, 36] signatures have been considered; the sample size selected for each random series provides a maximum error of 5% with 95% confidence in the average of Hamming distances. SBLs from 2 to 30 were considered for every digital signature. Thus, results for signature hosting through sum as combinational logic are shown in Table 1, while Table 2 resumes the hosting results for linear combination as combinational logic. Each row shows the results for the automated hosting of the number of digital signatures necessary to ensure a maximum error of ±1 with a 95% confidence, under the developed algorithms. For every automated algorithm and SBL, these tables contain measurements of the average Hamming distance (HDA) for the input patterns obtained as signature locations and the computation time (CT) of the corresponding algorithm.

In general, the three hosting algorithms get to significantly reduce the time required by a manual hosting. In order to evaluate this time effort reduction, let us consider a real manual hosting, for example, the manual hosting made for generating IPP designs included in [17], taking 8-bit blocks of MD5 and SHA1 digital signatures (DiTEC, UGR, and FSU) and using linear combination as combinational logic. As (4) shows, each signature bit block is decomposed as the sum of two products, existing different possibilities to generate the same signature block. An optimistic estimation gives at least 2^10^ different signature hosting possibilities for each signature block. In addition, taking into account the number of 8-bit blocks of MD5 and SHA1 signatures, the total number of different hosting possibilities for each digital signature is at least (2^10^)^16^. As the manual exploration of all hosting possibilities was not possible, the manual hosting was based in a human selection of the possibilities with apparently reduced HDA. For each digital signature, it took around 12 hours to evaluate the selected hosting possibilities in terms of HDA to finally choose one of them. Table 2 reflects that the optimized signature hosting of 8-bit blocks of MD5 digital signature using EPO and SAMB algorithms takes around 2.6 and 6.6 seconds, respectively.

Thus, it is confirmed that these hosting algorithms get an important reduction of the time effort exploring many more hosting possibilities than the manual hosting and getting less area overhead, as it will be shown in Section 3. At the following a more exhaustive analysis of each one of the algorithms in terms of computation time and HAD is made.

**Figure 3:**
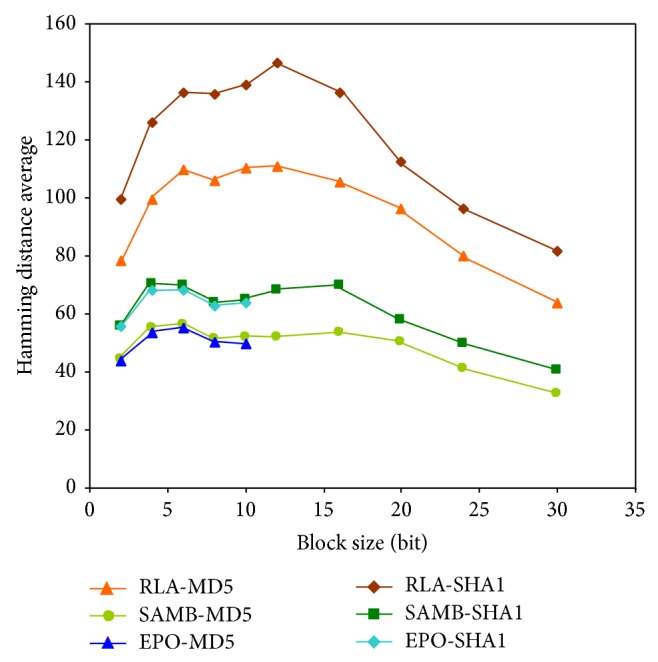
HDA for RLA and SAMB algorithms.

The main advantage of the RLA algorithm, besides automating the signature hosting, is the low computation times observed. However, the HDA data reflect that this algorithm does not offer the optimum solution for signature hosting. On the other hand, the EPO algorithm gets to reduce significantly the HDAs when compared to the RLA algorithm, but it requires excessive computation time for SBLs larger than 10 bits. Table 1 and Table 2 also display the main advantages of SAMB algorithm compared to the other two algorithms. First, SAMB gets to reduce the HDA, especially when compared to the RLA algorithm.  Figure 3 displays HDA data from Table 1. As it can be observed, SAMB and EPO get to reduce this parameter compared to RLA, thus improving the additional logic for signature extraction in terms of area increase.

**Figure 4:**
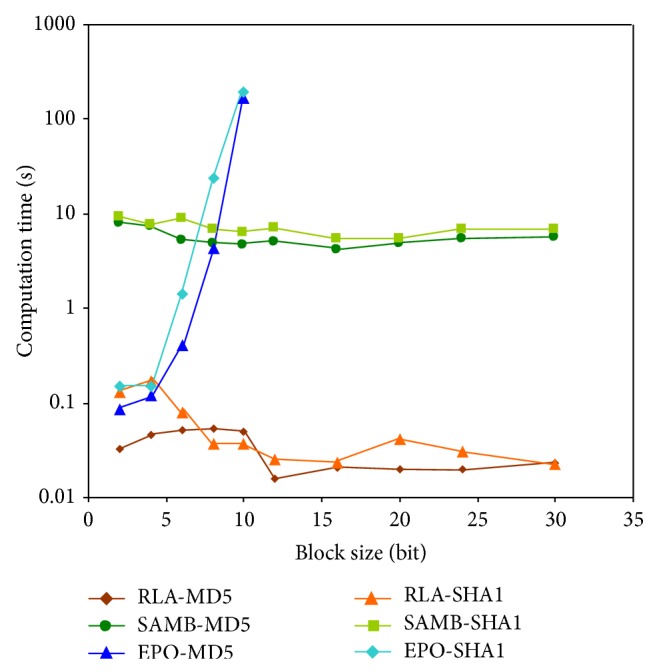
Computation time for EPO and SAMB algorithms.

Additionally, SAMB algorithm also has important advantages over the EPO algorithm when computation times are analyzed. For small block lengths, computation times of the SAMB algorithm are higher than those obtained for the EPO algorithm. For example, in the case of the sum, for the smallest block length, 2 bits, the computation time is 0.084 and 8.04 seconds, respectively, as Table 1 shows. However, for a 10-bit block length, the computation time for EPO is 166.83 seconds, while for SAMB it is just 4.56 seconds. It is observed that, as the block length increases, the computation times of the SAMB algorithm are considerably reduced compared to those of EPO. In general, for block lengths greater than 10 bits, the computation times for EPO are too long, while for the SAMB algorithm computation times are in the same range for all block lengths. These computation times for EPO algorithm are shown in Tables 1 and 2 as resources exceeded. Computation times obtained for the sum have been used to develop the graph shown in Figure 4. The trend of the curve for the EPO algorithm is exponential, with unacceptable values for the considered application. However, for the SAMB and RLA algorithms, the data are located within a range under 5 and 0.2 seconds, respectively, even for SBL sizes larger than 10 bits, whose computation times are at least 2 orders of magnitude lower than those obtained for the EPO algorithm. Thus, it can be concluded that the EPO algorithm is the best option for automating searches up to 10-bit SBLs, since in general it achieves best results for the minimization of the Hamming distance, as Tables 1 and 2 and Figure 3 reflect. On the other hand, for larger SBLs, the SAMB algorithm gets to minimize the Hamming distance with computation times significantly lower than those for EPO as Figure 4 displays. The RLA algorithm could be useful when the SBL is so large that makes the computation time excessive, even for SAMB (over 40 bits).

Summarizing this analysis, the SAMB algorithm results in important advantages, since it gets to automate signature hosting, to select the signature block length, and to minimize the HDA when compared to the random assignment, while maintaining reasonable CTs as the SBL size grows.

## 3. Results and Discussion

As it has been discussed above, the developed tool automates the signature hosting process, reducing the required effort and time when compared to a manual hosting. For illustrating the benefits of the new tool and its optimization related to the extraction logic resources, SL automated searches for MD5 and SHA1 digital signatures were carried out for the 8-tap programmable 1D-DWT filter bank detailed in [17], with 16-bit input and 19-bit output, including polyphase filter decomposition. The system is programmable in the sense that it has to be initialized by loading the corresponding filter coefficients according to the sequence to be computed. The system is based on the standard architecture for FIR filters [30]. The total system exhibits symmetry for the computation of the approximation and detail sequences. This 1D-DWT core has been used to make an exhaustive analysis of the three developed algorithms (RLA, SAMB, and EPO), considering different signatures (“DiTEC,” “UGR,” and “FSU”), different signature lengths (MD5, SHA1), and signature extraction logic (FSM, LFSR). Because of the structure of the system, the possibility to host every two 8-bit signature bit blocks as the addition of two linear combinations was considered. To generate the 1D-DWT IPP designs, the three algorithms were used separately for signature hosting purposes. In order to show that the results do not depend neither on the target technology nor on the design flow, Xilinx devices (along with ISE Project Navigator tools) and Altera devices (using Quartus II tools) were targeted.  Table 3 and Table 4 show the area resources for the IPP DWT designs, also detailing the implementation resources required for the FSMs in the signature extraction additional logic of each IPP design. These tables also show the HD between input patterns of every signature hosting. The maximum frequency for the watermarked cores is not shown because throughput penalization is almost negligible, less than 1% for all the IPP designs.  Table 3 resumes results using Xilinx device families, Spartan 3, and Virtex 5, for three different MD5 and SHA1 digital signatures (DiTEC, UGR, and FSU). On the other hand, Table 4 details the obtained data for SL automated searches for the same digital signatures using a different Altera device family, concretely Apex20KC. It must be noted that the main objective of these synthesis results is to show how the application of automated IPP@HDL affects the generic logic, without including any other element such as custom silicon blocks that the device family might include. In this way, Apex20KC was selected for Altera synthesis examples because this device family only includes logic elements and memory and does not incorporate custom silicon blocks, in contrast to most modern device families. Comparing these device families, when using Apex20KC, it is possible to keep software tools from taking some uncontrolled actions when the user indicates that only generic logic has to be used for synthesis and implementation. In addition, the new Apex20KC synthesis results for automated signature hosting allow comparing them to Apex20KC synthesis results for manual signature hosting in [17].

As Table 2 shows, for the linear combination and 8-bit signature bit blocks, the EPO algorithm should generate better hosting results, since it reduces the cost function, that is, the Hamming distance, while, for this signature block length, it does not require excessive computation time. Analyzing HD columns in Table 3 and Table 4, it can be seen that signature hosting for EPO algorithm achieves the lowest values of HD, which was predictable according to Table 2. Because of the more exhaustive HD minimization performed by the EPO algorithm, the FSM columns show better figures for this algorithm compared to the RLA and, in general, to the SAMB algorithm as well. It is because, as it was pointed in Section 2.2, this HD minimization contributes strongly to reducing the additional logic required by the FSM. Thus, EPO and SAMB algorithms, in addition to automating the signature hosting process, reduce area overhead when compared to the RLA algorithm, confirming that the selected cost function gets this initial objective. The HD and implementation results obtained for the RLA algorithm are similar to those obtained for manual hosting [17], but by using this RLA algorithm the signature hosting process is automated, saving time and effort when compared to a manual signature insertion. Thus, it is possible to initially evaluate the improvements that can be found with the optimized automated hosting: automation and hosting optimization. IPP designs and FSMs using EPO and SAMB algorithms in the automated search achieve similar area results that are, in general, much lower than those for RLA.

In some cases, the use of the SAMB algorithm achieves area results even better than EPO. There are several reasons for this. First, the HDs for both EPO and SAMB are very close, or, in some cases, equal. Second, the FSM resources do not only depend on the selected cost function (HD) and the FSM optimization, but also depend on the overall design implementation. When the HDs of two different signature hosting options are close, the dependency on other parameters is slightly noticeable, while, in general, its area supposes less than 0.5% over the total area implementation of IPP designs. Thus, the HD between the input patterns that address the signature bit blocks as cost function gets a good optimization of the signature hosting automation. Finally, internal issues of the design tools for synthesis and logic optimization have to be considered. The implementation results of Tables 3 and 4 show that both EPO and SAMB optimize the hosting process; thus, additional resources are minimized. These results confirm that the selection of the Hamming distance between the input patterns generating the signature blocks as cost function reduces the area overhead of the watermarked cores. In addition, these results also ratify that SAMB is a good signature hosting alternative to ensure reduced computation times and the minimization of the area increase of the watermarked IP cores.

Comparing Altera results shown in Table 3 to Altera results in [17], where the signature hosting was manually performed, there is a reduction of the area penalization for the IPP designs generated by the automated tool. For example, for 1D-DWT FSM design and MD5 digital signature, [17] shows an area increase of 1.7%, while the new synthesis results show that the use of the automated tool reduces the area increase down to 1.2% for the SAMB algorithm (6863 LEs for the original 1D-DWT design). This reduction of the area penalty is caused by the fact that the use of the automated tool for SL selection leads to reduced FSM implementations, due to the minimization of HDs. Compared to a manual search, the automated algorithms achieve important time and effort savings, while also reducing area penalization.

The new tool for signature distribution, applied to IPP@HDL, preserves the structure and initial benefits of IPP@HDL, while it automates the application of this watermarking technique. As it has been mentioned in previous sections, this contributes to a considerable reduction of time and effort required for the generation of the watermarked core. It has been tested by means of manual and automated hosting. While manual hosting could require long hours of works for an expert designer, the computation time needed for automated hosting is reduced to just mere seconds. It is important to remember that automated hosting also allows optimizing the signature hosting maintaining this computation time reduction.

## 4. Conclusions

IPP@HDL method has proven to be a valuable method for watermarking HDL designs. However, the design process so far has been tedious and labor intensive requiring to manually insert the signatures into the HDL design. This paper introduces custom tools for easing the signature hosting and limiting manual design as much as possible. Three algorithms were developed for IPP@HDL automated signature distribution: RLA, EPO, and SAMB algorithms. The tool for signature hosting can noticeably reduce the area penalty associated to the application of IPP@HDL, while eliminating the need of manual signature insertion that may require long hours of work. In order to illustrate this, exhaustive data for several signatures have been obtained using the RLA, EPO, and SAMB algorithms. Results show the benefits of SAMB. This SAMB algorithm results in important advantages, since it minimizes the average Hamming distance when compared to the random assignment, while maintaining reasonable computation times as the signature block location sizes grow. The impact of the tool in the hardware implementation is also illustrated through the synthesis on field-programmable logic of a series of 1D-DWT cores using the signature locations provided by the automated algorithms. From the presented data, it is evident that the benefits of the original watermarking technique are improved, while the signature hosting process is easily and automatically carried out, saving significant design time and effort while the area overhead is minimized. The developed tool can also be used for different applications trying to solve optimization problems on the distribution of signature bits for IP core protection.

## Figures and Tables

**Algorithm 1:**
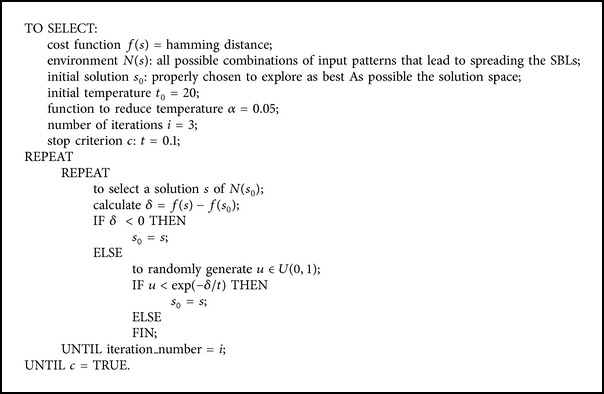
Scheme for simulated annealing algorithm.

**Table 1:** HDAs and CTs (PentiumIV at 2.2 GHz) with the automated tool for the sum as combinational logic (^*^resources exceeded).

DS	SBL	RLA		EPO		SAMB	
		HDA	CT (s)	HDA	CT (s)	HDA	CT (s)
MD5	2	78	0.032	43	0.084	44	8.04
	4	99	0.046	53	0.120	55	7.25
	6	109	0.051	55	0.410	56	5.16
	8	106	0.053	50	4.280	51	4.74
	10	110	0.050	49	166.8	52	4.56
	12	110	0.016	∗	∗	52	5.08
	16	105	0.021	∗	∗	53	4.20
	20	96	0.020	∗	∗	50	4.77
	24	80	0.020	∗	∗	41	5.30
	30	64	0.023	∗	∗	32	5.70

SHA1	2	99	0.130	55	0.150	55	9.11
	4	125	0.170	68	0.150	70	7.71
	6	136	0.078	67	1.410	69	8.89
	8	135	0.037	62	23.90	63	6.77
	10	139	0.036	63	195.9	64	6.31
	12	146	0.025	∗	∗	67	7.15
	16	136	0.023	∗	∗	69	5.40
	20	112	0.041	∗	∗	57	5.43
	24	96	0.030	∗	∗	49	6.83
	30	81	0.022	∗	∗	40	6.90

**Table 2:** HDAs and CTs (PentiumIV at 2.2 GHz) with the automated tool for the linear combination as combinational logic (^*^resources exceeded).

DS	SBL	RLA		EPO		SAMB	
		HDA	CT (s)	HDA	CT (s)	HDA	CT (s)
MD5	2	99	0.06	24	0.7	25	5.2
	4	110	0.02	32	0.6	34	3.8
	6	123	0.01	54	0.2	59	4.7
	8	119	0.01	47	2.6	50	6.6
	10	117	0.01	40	54	46	7.0
	12	118	0.03	∗	∗	46	12
	16	112	0.15	∗	∗	51	35
	20	113	0.13	∗	∗	59	156
	24	120	1.16	∗	∗	65	383
	30	120	11.0	∗	∗	70	2000

SHA1	2	122	0.09	30	0.3	32	6.7
	4	136	0.02	41	0.4	42	4.9
	6	152	0.01	66	0.2	72	6.0
	8	149	0.03	65	5.3	64	7.4
	10	148	0.02	50	152	57	9.2
	12	155	0.05	∗	∗	61	18
	16	142	0.26	∗	∗	64	44
	20	139	0.95	∗	∗	68	103
	24	143	1.09	∗	∗	77	730
	30	150	9.08	∗	∗	88	4200

**Table 3:** Detailed synthesis results for Xilinx design tools, Spartan III, and Virtex 5 families.

IPP designs	DS	Spreading	HD			Spartan 3				Virtex 5		
				SLICEs	4 input LUTs	FSM		SLICEs	LUT Flip Flop pairs	DSP	FSM	
						SLICEs	4 input LUTs				SLICEs	LUT Flip Flop pairs
1D-DWT FSM	DiTEC MD5	RLA	91	3017	5292	18	29	897	3337	7	16	27
		SAMB	36	3015	5289	17	**27**	891	3303	7	14	25
		EPO	30	3014	5287	16	25	888	3317	7	13	23
	DiTEC SHA1	RLA	102	3023	5303	25	41	905	3329	7	29	39
		SAMB	52	3019	5295	19	32	889	3285	7	19	30
		EPO	46	3017	5293	19	31	889	3307	7	17	28
	UGR MD5	RLA	89	3017	5292	18	**29**	897	3329	7	17	27
		SAMB	37	3015	5289	16	**26**	881	3282	7	14	24
		EPO	33	3015	5290	17	27	889	3317	7	14	25
	UGR SHA1	RLA	102	3024	5305	26	43	900	3334	7	30	38
		SAMB	49	3013	5290	16	30	895	3315	7	20	24
		EPO	49	3019	5295	21	34	891	3307	7	20	31
	FSU MD5	RLA	93	3017	5292	18	**29**	897	3377	7	16	27
		SAMB	41	3013	5289	16	**24**	880	3284	7	14	24
		EPO	33	3014	5289	16	26	883	3291	7	13	24
	FSU SHA1	RLA	106	3022	5302	23	40	898	3320	7	25	37
		SAMB	41	3018	5295	21	33	894	3308	7	21	31
		EPO	37	3018	5292	20	32	892	3311	7	19	29

1D-DWT LFSR	DiTEC MD5	RLA	91	3032	5317	9	13	**908**	**3370**	7	13	16
		SAMB	36	3030	5314	8	11	**898**	**3341**	7	11	14
		EPO	30	3029	5312	7	9	897	3332	7	8	12
	DiTEC SHA1	RLA	102	3038	5329	15	24	918	3370	7	24	28
		SAMB	52	3033	5319	11	16	901	3345	7	14	19
		EPO	46	3032	5319	11	16	900	3344	7	15	20
	UGR MD5	RLA	89	3032	5317	9	13	916	3362	7	13	16
		SAMB	37	3030	5314	8	11	902	3347	7	10	13
		EPO	33	3030	5315	8	11	905	3357	7	11	14
	UGR SHA1	RLA	102	3039	5330	16	26	917	3379	7	24	29
		SAMB	49	3033	5321	12	18	908	3358	7	17	21
		EPO	49	3034	5320	11	17	907	3358	7	16	20
	FSU MD5	RLA	93	3032	5317	9	13	900	3340	7	12	16
		SAMB	41	3028	5314	8	10	893	3335	7	10	13
		EPO	33	3029	5314	8	11	897	3335	7	9	12
	FSU SHA1	RLA	106	3037	5327	14	23	914	3377	7	17	26
		SAMB	41	3032	5320	11	17	901	3346	7	15	20
		EPO	37	3033	5318	10	14	899	3332	7	14	18

**Table 4:** Detailed synthesis results for Altera design tools and Apex20kc family.

IPP designs	DS	Spreading	HD	LEs	FSM LEs
1D-DWT FSM	DiTEC MD5	RLA	**91**	6965	41
		SAMB	**36**	6947	37
		EPO	30	6963	36
	DiTEC SHA1	RLA	102	6962	41
		SAMB	52	6945	33
		EPO	46	6940	31
	UGR MD5	RLA	**89**	6969	41
		SAMB	**37**	6966	35
		EPO	33	6966	35
	UGR SHA1	RLA	102	6960	43
		SAMB	49	6943	35
		EPO	49	6940	34
	FSU MD5	RLA	**93**	6971	41
		SAMB	**41**	6949	37
		EPO	33	6964	37
	FSU SHA1	RLA	106	6963	40
		SAMB	41	6935	34
		EPO	37	6935	32

1D-DWT LFSR	DiTEC MD5	RLA	**91**	6995	19
		SAMB	36	6981	15
		EPO	30	6974	14
	DiTEC SHA1	RLA	102	7050	29
		SAMB	52	7018	20
		EPO	46	7007	21
	UGR MD5	RLA	89	**7026**	**18**
		SAMB	**37**	6985	15
		EPO	33	6981	14
	UGR SHA1	RLA	102	7042	31
		SAMB	49	7013	23
		EPO	49	7003	19
	FSU MD5	RLA	**93**	**7009**	**18**
		SAMB	**41**	6985	15
		EPO	33	6980	15
	FSU SHA1	RLA	106	7041	29
		SAMB	41	6999	21
		EPO	37	6996	19
